# When less is more: Gaining power through gene rearrangement of amplified *EGFR*

**DOI:** 10.18632/oncotarget.26786

**Published:** 2019-03-15

**Authors:** Tomoyuki Koga, Clark C. Chen, Frank B. Furnari

**Affiliations:** Ludwig Cancer Research, University of California San Diego, La Jolla, CA, USA

**Keywords:** genetic rearrangement, mutation, oncogene amplification

Extrachromosomal gene amplification refers to copy number gains of DNA fragments that are not contiguous with the twenty-three canonical chromosomes. Unlike typical chromosomes, they are composed of circular DNA fragments without centromeric sequences [1]. They arose through complex genomic rearrangements during carcinogenesis and commonly harbor oncogenes, conferring selective advantage for tumor growth and survival [2]. Such extrachromosomal elements, often referred to as double minutes, are frequently observed throughout different types of cancer, especially in glioblastoma [3]. They are dynamically regulated and play important roles in acquired therapeutic resistance [4] and clonal evolution [3].

Epidermal growth factor receptor gene (*EGFR*) is, by far, the most commonly altered oncogene in glioblastomas, the most common form of primary brain cancer. It is mutated or amplified in 60% of glioblastomas [5]. Oncogenic activation of EGFR appears to preferentially occur through deletion of exons two to seven, generating a constitutively activated receptor termed EGFRvIII. Up to 30% of glioblastomas harbor EGFRvIII. Importantly, EGFRvIII is always accompanied by amplification of the full-length *EGFR* gene. EGFRvIII-positive glioblastomas exhibit unique molecular physiologies associated with distinct therapeutic response profiles and an overall tumor aggressiveness [6].

Breakpoint junctions in EGFRvIII are highly variable between tumor samples due to 120-kb-long intron one of this gene where one of the breaks take place. Breakpoint sequence analyses implicate contribution by multiple DNA strand-break repair mechanisms, including non-homologous end joining, micro-homology mediated end joining, and other forms of replication mediated repair [7]. These findings suggest DNA strand breaks as a likely intermediate structure for the *EGFR* deletion variants.

**Figure 1:**
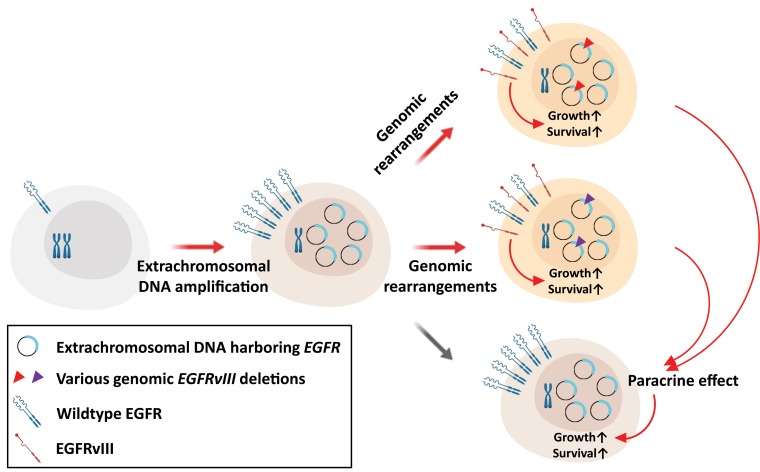
The possible mechanisms of tumor progression through genomic rearrangements of extrachromosomal DNA in glioblastoma Models for generation of heterogeneous *EGFRvIII* breakpoints through genomic rearrangements. Cells with extrachromosomal amplifications of *EGFR* undergo further independent genomic rearrangements resulting in heterogeneous populations expressing EGFRvIII from differentially edited *EGFR* genes. Those cells acquiring *EGFRvIII* are not only enhanced for aggressive tumor growth but also benefit surrounding cells without the *EGFR* mutation through paracrine effect.

Another interesting observation pertaining to *EGFRvIII* is that a single tumor can harbor multiple, independent *EGFRvIII* variants -- each with distinct breakpoint junctions. These variants as well as the accompanied amplified full-length EGFR share common allelic profiles, suggesting that the deleted variants arose from a common ancestral amplified *EGFR*. A corollary of this hypothesis is that distinct additional genomic rearrangements of amplified full-length *EGFR* in glioblastoma subpopulations gave rise to polyclonal *EGFRvIII* breakpoint species (Figure 1).

An important mechanistic question in this context involves the sequence of events that led to *EGFR* variant formation and *EGFR* amplification. Is extrachromosomal *EGFR* amplification a requisite precursor of *EGFRvIII?* For instance, this acquisition of glioblastoma specific *EGFR* deletion mutation might be associated with the fact that amplified *EGFR* in glioblastoma mostly resides in extrachromosomal DNA that is selectively captured in micronuclei [1], where complex DNA rearrangements take place [8]. In this way, extrachromosomal DNA generation possibly provides a path for the host cell to enhance malignant progression through micronuclei-mediated genomic rearrangements resulting in *EGFRvIII*.

Once *EGFRvIII* is formed, not only do cells expressing this mutant transform to a more aggressive phenotype as mentioned above, but surrounding tumor cells without mutant *EGFR* are also benefited in terms of growth and cell survival by paracrine effects through cytokines such as interleukin-6 [9], or through EGFR ligands such as heparin-binding EGF and transforming growth factor alpha [10] (Figure 1). Thus, tumors with subpopulations undergoing genomic rearrangements of amplified *EGFR* yielding *EGFRvIII* that establishes an ecosystem supportive of aggressive tumor growth. Elucidation of this mechanisms of further rearrangements of extrachromosomal amplicons should yield opportunities for future therapeutic development and mechanistic insight.
